# Right ventricular outflow tract aneurysm as an incidental finding in a patient with acute pericarditis: A case report

**DOI:** 10.1016/j.radcr.2025.12.048

**Published:** 2026-01-30

**Authors:** Seyed Reza Tabibian, Faezeh Tabesh, Farshad Riahi

**Affiliations:** a Radiologist, School of Medicine, Isfahan university of medical sciences, Isfahan, Iran; b Interventional Cardiology Research Center, Cardiovascular Research Institute, Isfahan University of Medical Sciences, Isfahan, Iran; c Department of Radiology, Isfahan University of Medical Sciences, Isfahan, Iran

**Keywords:** Right ventricular outflow tract aneurysm, Acute pericarditis, Multidetector computed tomography, Incidental finding, Conservative management, Cardiac imaging

## Abstract

Right ventricular outflow tract (RVOT) aneurysms are rare, often linked to congenital defects or surgery, and their incidental detection during acute pericarditis is unreported. This case highlights the diagnostic role of imaging in atypical presentations. A 32-year-old male presented with a 2-week history of sharp, pleuritic chest pain worsened by inspiration and coughing, partially relieved by nonsteroidal anti-inflammatory drugs (NSAIDs). He had a 10 pack-year smoking history but no prior cardiac disease. Physical examination was unremarkable. Laboratory tests showed mildly elevated C-reactive protein (CRP) and erythrocyte sedimentation rate (ESR). Electrocardiography (ECG) was normal, but transthoracic echocardiography revealed a left ventricular ejection fraction (LVEF) of 40%, right ventricular enlargement, and moderate pericardial effusion. Multidetector computed tomography (MDCT) with pulmonary thromboembolism (PTE) protocol excluded PTE but incidentally identified RVOT aneurysm. Conservative management with aspirin and colchicine led to symptom resolution; at 6-month follow-up, the patient was asymptomatic with stable imaging. This incidental finding underscores MDCT’s utility in detecting coexisting structural anomalies in patients presenting with pericarditis. Conservative management is appropriate for asymptomatic cases without obstruction.

## Introduction

Right ventricular outflow tract (RVOT) aneurysms are exceedingly rare cardiac anomalies, with an estimated incidence of less than 1% in patients undergoing congenital heart disease repair or in those with idiopathic etiologies [1]. They typically arise from congenital defects, prior surgical interventions (eg, tetralogy of Fallot repair), trauma, or infections, leading to focal dilation of the RVOT wall [2]. Complications may include obstruction, thrombosis, or rupture; however, many are asymptomatic and are discovered incidentally [3].

Acute pericarditis, characterized by inflammation of the pericardial sac, presents with pleuritic chest pain, effusion, and tamponade [4]. While pericarditis is common (affecting ∼0.1%-0.2% of hospitalized patients) [5], its intersection with RVOT aneurysms is exceptionally rare and, to our knowledge, unreported. Possible mechanisms include inflammatory weakening of the RVOT wall, akin to processes in aortic aneurysms, or shared etiologies, such as viral triggers [6,7]. This association poses diagnostic challenges, as pericardial symptoms may mask underlying structural issues of the heart.

This case report documents a spontaneous RVOT aneurysm incidentally found in a young adult with acute pericarditis without typical risk factors. By highlighting this novel presentation, we aim to emphasize the role of advanced imaging in the differential diagnosis and advocate for tailored management strategies.

## Case presentation

A 32-year-old man presented to the emergency department with a 2-week history of sharp, non-radiating chest pain localized to the anterior thorax. The pain was pleuritic in nature, exacerbated by deep inspiration, coughing, and supine positioning, with partial relief when sitting forward. He reported mild improvement with nonsteroidal anti-inflammatory drugs (NSAIDs) but denied associated symptoms including dyspnea on exertion, palpitations, orthopnea, paroxysmal nocturnal dyspnea, syncope, or fever.

His medical history was unremarkable with no prior cardiovascular disease, autoimmune conditions, or recent infections. He had no history of cardiac surgery, chest trauma, or invasive cardiac procedures. Furthermore, a review of the patient’s medical records confirmed that no prior thoracic or cardiac imaging had been performed, leaving the chronicity and potential congenital origin of the RVOT aneurysm to be inferred from its current presentation. Social history revealed a 10 pack-year smoking history but no alcohol or illicit drug use. Family history was negative for sudden cardiac death or structural heart disease.

On physical examination, the vital signs were stable (blood pressure, 118/76 mmHg; heart rate, 82 bpm; respiratory rate 16/min, oxygen saturation, 98% on room air). There was no pericardial rub, jugular venous distension, or respiratory distress noted. Cardiac auscultation revealed normal heart sounds, without murmurs.

Laboratory investigations showed mild inflammation: CRP 15 mg/L (normal <5 mg/L) and ESR 25 mm/h (normal <20 mm/h), and white blood cell count 11,200/μL (normal 4000-10,000/μL). Complete metabolic panel, including electrolytes, renal function, and liver enzymes, was within normal limits. Cardiac biomarkers including troponin I (<0.01 ng/mL) and creatine kinase-MB were not elevated. Thyroid function tests and urinalysis were normal.

**Fig. 1:**
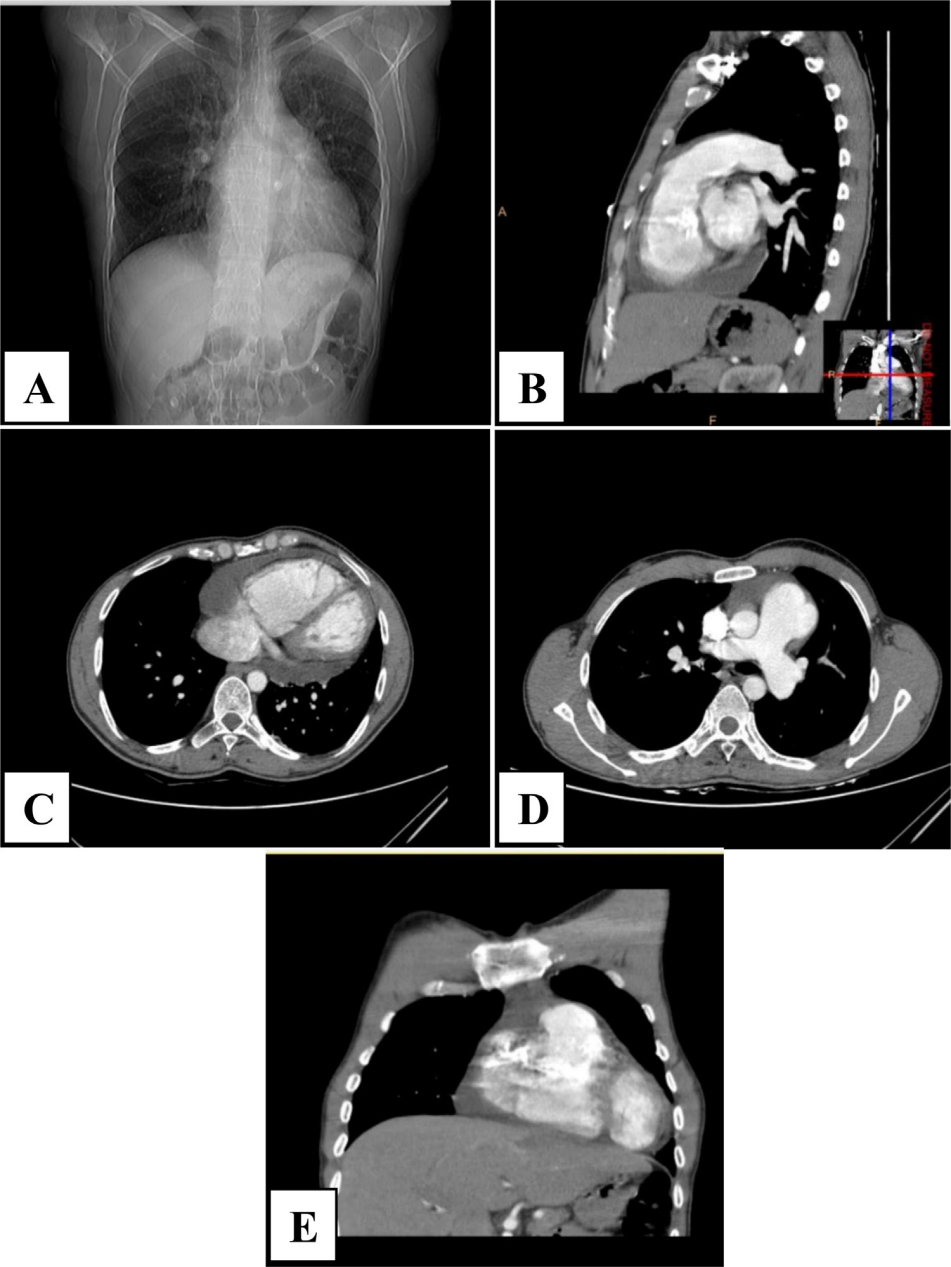
Pulmonary CT angiography: A, scout image; B, sagittal view; C, axial view; D, axial view; E, coronal view. Arrows in panels B, D, and E highlight the focal aneurysmal dilation of the right ventricular outflow tract.

Electrocardiography (ECG) showed normal sinus rhythm at 86 beats per minute with no ST-segment changes or other abnormalities suggestive of ischemia. Transthoracic echocardiography (TTE) revealed a left ventricular ejection fraction (LVEF) of 40%, significant right ventricular enlargement, and moderate pericardial effusion. A multidetector computed tomography (MDCT) scan with pulmonary thromboembolism (PTE) protocol was conducted to further assess the chest pain and exclude pulmonary embolism (Fig. 1). The imaging unexpectedly revealed a 3.5 × 4.2 cm thin-walled right ventricular outflow tract (RVOT) aneurysm. The wall appeared thin but continuous with the myocardial layers, suggesting a true aneurysm rather than a pseudoaneurysm. This was an uncommon incidental finding in a patient with acute pericarditis.

After confirming the diagnosis of a RVOT aneurysm and ruling out pulmonary embolism, the patient was treated conservatively and exhibited clinical improvement. The patient was discharged in stable condition, exhibiting stable vital signs and no evidence of hemodynamic compromise. A comprehensive discharge plan was provided, which included instructions for monitoring danger signs such as exacerbating chest pain, dyspnea, fever, or indications of cardiac tamponade. Pharmacologic therapy consisted of aspirin 325 mg, administered as 2 tablets every 8 hours for a duration of 10 days, and colchicine 0.5 mg, taken once daily for 2 months, aimed at addressing the inflammatory aspect associated with pericarditis. The patient was instructed to refrain from strenuous activities and was scheduled for an outpatient cardiology follow-up to assess cardiac function and monitor aneurysm progression.

## Discussion

RVOT aneurysms are rare cardiac anomalies that can be discovered incidentally in patients with acute pericarditis. These aneurysms are usually associated with prior cardiac surgery, trauma, infection, or congenital defects [1]. In cases of acute pericarditis, imaging may reveal unexpected structural anomalies, which can be distinguished using TTE, CT or cardiac magnetic resonance imaging (MRI) [8]. It is crucial to differentiate between true aneurysm and pseudoaneurysm due to differences in wall composition and rupture risk. A 2021 case report highlighted the importance of multimodal imaging for accurate diagnosis and risk stratification [9]. Management of RVOT aneurysms depends on size, symptoms, and risk of rupture. In pericarditis, the inflammatory environment may obscure or mimic aneurysmal changes. From a radiological perspective, significant pericardial effusion can compress or mask the external contours of the RVOT on transthoracic echocardiography (TTE). Clinically, the dominant symptoms of pleuritic pain may lead clinicians to overlook structural anomalies. Cross-sectional imaging, specifically MDCT or cardiac MRI, is essential in these cases to provide the high spatial resolution needed to differentiate pericardial fluid from true aneurysmal wall dilation.

Our case uniquely describes a spontaneous RVOT aneurysm incidentally detected in a 32-year-old male, presenting initially with pericarditis, an association that is, to our knowledge, unreported in the existing literature and not listed as a known complication. The patient’s presentation with pleuritic chest pain, elevated inflammatory markers, and moderate pericardial effusion, in the absence of trauma, prior cardiac surgery, or other predisposing conditions [10]. Potential mechanisms include pericardial inflammation causing RVOT wall degradation via cytokines (eg, IL-6, TNF-α), similar to inflammatory aneurysms [11,12]. The patient’s reduced LVEF (40%) and significant right ventricular enlargement, in the absence of elevated Troponin I (<0.01 ng/mL), are atypical for simple acute pericarditis. These findings suggest that the RVOT aneurysm and ventricular dysfunction may be components of an underlying chronic condition, such as Arrhythmogenic Right Ventricular Cardiomyopathy (ARVC) or a silent chronic cardiomyopathy, rather than acute myopericarditis. The lack of troponin elevation further supports a chronic process over acute myocardial inflammation, warranting serial monitoring and genetic counseling [13].

Diagnostic efforts, including TTE and MDCT, were crucial not only for identifying the unexpected RVOT aneurysm but also for ruling out pulmonary embolism, a common differential for the patient’s symptoms [6]. Unlike many reported cases where RVOT aneurysms cause significant obstruction requiring surgical intervention (often characterized by a right ventricle-pulmonary artery systolic pressure gradient ≥35 mmHg), our patient exhibited right ventricular enlargement but no evidence of severe RVOT obstruction or intra-aneurysmal thrombus, which are typical indications for re-operation [14]. This absence of severe mechanical complications supported a conservative management strategy with close follow-up, emphasizing the diverse clinical spectrum and management considerations for RVOT aneurysms.

In conclusion, the incidental discovery of a spontaneous right ventricular outflow tract aneurysm during an episode of acute pericarditis represents an exceptionally unusual clinical situation. This case highlights the significance of modern imaging techniques, such as MDCT, in detecting intricate cardiac abnormalities that coexist with unconventional primary symptoms. While the clinical presentation of pericarditis led to the discovery, the structural anomaly likely represents a chronic, independent condition. This case demonstrates that, without significant obstruction or thromboembolic risk, diligent monitoring and patient education are suitable; however, ongoing outpatient cardiology follow-up is essential to evaluate aneurysm progression and underlying cardiac function.

## Patient consent

Written informed consent was obtained from the patient.
